# Editorial: Impact of emerging treatment modalities on stromal cells in the tumour microenvironment

**DOI:** 10.3389/fimmu.2024.1454001

**Published:** 2024-07-02

**Authors:** Haiyang Wu, Dietrich Alexander Ruess, Jinxue Zhou, Haitao Zhao

**Affiliations:** ^1^ Department of Orthopaedics, The First Affiliated Hospital of Zhengzhou University, Zhengzhou, China; ^2^ Department of Clinical College of Neurology, Neurosurgery and Neurorehabilitation, Tianjin Medical University, Tianjin, China; ^3^ Department of General and Visceral Surgery, Center for Surgery, Medical Center University of Freiburg, Freiburg, Germany; ^4^ German Cancer Consortium (DKTK), Partner Site Freiburg and German Cancer Research Center (DKFZ), Heidelberg, Germany; ^5^ Department of Hepatobiliary and Pancreatic Surgery, The Affiliated Tumor Hospital of Zhengzhou University, Zhengzhou, China; ^6^ Department of Liver Surgery, State Key Laboratory of Complex Severe and Rare Diseases, Peking Union Medical College Hospital, Chinese Academy of Medical Sciences and Peking Union Medical College (CAMS & PUMC), Beijing, China

**Keywords:** stromal cells, tumor microenvironment, non-small cell lung cancer, premetastatic niches, immune checkpoint inhibitor

Traditional cancer therapies, including surgery, radiation, and chemotherapy, have long been the cornerstone in treating malignant tumors. However, with advancements in science and technology, novel cancer treatment modalities such as immunotherapy, targeted therapy, sonodynamic therapy, and photodynamic therapy have emerged, revolutionizing the therapeutic landscape. These innovative approaches not only transform the paradigms of tumor treatment but also significantly impact the stromal cells within the tumor immune microenvironment. Stromal cells, which are crucial components of the tumor microenvironment, include fibroblasts, endothelial cells, cancer stem cells and immune cells. These cells play pivotal roles in the initiation, progression, and metastasis of tumors. New therapeutic methods exert their effects on stromal cells through various mechanisms. For example, immunotherapy activates the patient’s own immune system, enabling immune cells to more effectively recognize and attack tumor cells while also altering the functions of tumor-associated fibroblasts, thus reshaping the tumor microenvironment. Sonodynamic and photodynamic therapies utilize specific wavelengths of sound and light waves to activate particular drugs within tumor cells, and then generate reactive oxygen species that kill the cancer cells. These methods also affect stromal cells by disrupting their interactions with tumor cells, thereby further inhibiting tumor growth and spread. Therefore, novel cancer treatments target not only tumor cells directly but also modulate stromal cells in the tumor microenvironment through multiple pathways, to achieve more effective anti-tumor outcomes. The application of these new therapies heralds a new era in cancer treatment and offers patients greater hope and possibilities. This Research Topic entitled “impact of emerging treatment modalities on stromal cells in the tumor microenvironment”, compiled work by authors from various research teams.

The first paper of the Research Topic was a review. Overcoming resistance to targeted therapy and immunotherapy in non-small cell lung cancer (NSCLC) remains a formidable obstacle in the treatment landscape. The resistance mechanisms are complex and multifaceted, involving alterations in molecular targets, activation of alternative signaling pathways, tumor heterogeneity, changes within the tumor microenvironment, immune evasion, and immunosuppression. Addressing these challenges requires a multi-pronged approach, including the development of combination therapies, a deeper understanding of resistance mechanisms to optimize the use of new drug targets, identification of relevant biomarkers, and modulation of the tumor microenvironment. In this review, Xiang et al., provide an overview of the diverse mechanisms contributing to resistance in NSCLC and explore the latest promising strategies aimed at overcoming these challenges to enhance treatment efficacy for NSCLC patients.

The second paper of the Research Topic was also a review related to pre-metastatic niches (PMNs) (Li et al.). Evidence indicates that extracellular vesicles (EVs) secreted by cancer cells play a critical role in orchestrating the development of PMNs. These tumor-derived EVs facilitate bidirectional communication between cancerous and stromal cells within both local and distant microenvironments. The EVs, which contain mRNAs, small RNAs, microRNAs, DNA fragments, proteins, and metabolites, influence metastatic organotropism, promote angiogenesis, alter stromal cell phenotypes, remodel the extracellular matrix, induce immunosuppression, and modify the metabolic landscape of organs. This review offers an in-depth analysis of PMNs formation and the mechanisms driven by EVs, along with potential strategies to inhibit cancer metastasis by targeting the formation of PMNs.

The third study focused on the role of monocytes in the tumor microenvironment to inform targeted cancer therapies (Li et al.). Using an innovative method with four 20-color flow cytometry panels, researchers conducted a comprehensive analysis of peripheral immune cells, especially monocyte subsets. Advanced techniques such as tSNE and FlowSom were used to profile 50 cell surface markers related to immunological functions. The results enhance the identification of monocyte subsets, aiding the development of personalized immunotherapies and improving diagnostic accuracy. Key discoveries include distinct marker expression patterns associated with tumor progression, offering new targets for therapeutic intervention.

The last study was a retrospective study that investigated the relationship between smoking history and the effectiveness of immune checkpoint inhibitors (ICIs) in bladder cancer patients (Kong et al.). Analyzing data from 348 patients, including a validation cohort of 248, the study examines smoking history, clinical characteristics, and immune profiles. While no significant differences in overall survival were found among current, former, and never smokers, former smokers exhibited a trend toward better immunotherapy responses. Additionally, PD-L1 expression was higher in former smokers. These findings suggest that smoking history may influence tumor response to ICIs, emphasizing the need to consider lifestyle factors in personalized cancer treatment strategies.

In conclusion, this Research Topic stresses the importance of stromal cells in the tumor microenvironment for cancer treatments. Key studies include overcoming resistance in NSCLC, the role of extracellular vesicles in pre-metastatic niche formation, monocyte profiling for personalized immunotherapies, and the influence of smoking history on immune checkpoint inhibitor effectiveness in bladder cancer. These advancements hold promise for more effective and personalized cancer treatments, highlighting the need for further exploration of the tumor microenvironment and patient-specific factors.

## Author contributions

HW: Conceptualization, Supervision, Validation, Writing – original draft, Writing – review & editing. DR: Conceptualization, Supervision, Validation, Writing – review & editing. JZ: Supervision, Writing – review & editing. HZ: Supervision, Writing – review & editing.

